# Quantifying primaquine effectiveness and improving adherence: a round table discussion of the APMEN Vivax Working Group

**DOI:** 10.1186/s12936-018-2380-8

**Published:** 2018-06-20

**Authors:** Kamala Thriemer, Albino Bobogare, Benedikt Ley, Clarice Samo Gudo, Mohammad Shafiul Alam, Nick M. Anstey, Elizabeth Ashley, J. Kevin Baird, Charlotte Gryseels, Elodie Jambert, Marcus Lacerda, Ferdinand Laihad, Jutta Marfurt, Ayodhia Pitaloka Pasaribu, Jeanne Rini Poespoprodjo, Inge Sutanto, Walter R. Taylor, Christel van den Boogaard, Katherine E. Battle, Lek Dysoley, Prakash Ghimire, Bill Hawley, Jimee Hwang, Wasif Ali Khan, Rose Nani Binti Mudin, Maria Endang Sumiwi, Rukhsana Ahmed, M. M. Aktaruzzaman, Kiran Raj Awasthi, Azucena Bardaji, David Bell, Leonard Boaz, Faustina Helen Burdam, Daniel Chandramohan, Qin Cheng, Keobouphaphone Chindawongsa, Janice Culpepper, Santasabuj Das, Raffy Deray, Meghna Desai, Gonzalo Domingo, Wang Duoquan, Stephan Duparc, Rustini Floranita, Emily Gerth-Guyette, Rosalind E. Howes, Cecilia Hugo, George Jagoe, Elvieda Sariwati, Sanya Tahmina Jhora, Wu Jinwei, Harin Karunajeewa, Enny Kenangalem, Bibek Kumar Lal, Chandra Landuwulang, Emmanuel Le Perru, Sang-Eun Lee, Leo Sora Makita, James McCarthy, Asrat Mekuria, Neelima Mishra, Esau Naket, Simone Nambanya, Johnny Nausien, Thang Ngo Duc, Thuan Nguyen Thi, Rinitis Noviyanti, Daniel Pfeffer, Gao Qi, Annisa Rahmalia, Stephen Rogerson, Iriani Samad, Jetsumon Sattabongkot, Ari Satyagraha, Dennis Shanks, Surender Nath Sharma, Carol Hopkins Sibley, Ali Sungkar, Din Syafruddin, Arunansu Talukdar, Joel Tarning, Feiko ter Kuile, Suman Thapa, Minerva Theodora, Tho Tran Huy, Edward Waramin, Govert Waramori, Adugna Woyessa, Chansuda Wongsrichanalai, Nguyen Xuan Xa, Joon Sup Yeom, Lukas Hermawan, Angela Devine, Spike Nowak, Indra Jaya, Supargiyono Supargiyono, Koen Peeters Grietens, Ric N. Price

**Affiliations:** 1 Global and Tropical Health Division, Menzies School of Health Research and Charles Darwin University, Darwin, Darwin, NT 0810 Australia; 2 Ministry of Health and Medical Services, National Vector-Borne Disease Control Programme, Honiara, Solomon Islands; 3 Independent Consultant, Amsterdam, The Netherlands; 4 International Center for Diarrheal Diseases (ICDDR,B), Dhaka, Bangladesh; 5 Myanmar-Oxford Clinical Research Unit, Yangon, Myanmar; 6 Centre for Tropical Medicine and Global Health, University of Oxford, Oxford, UK; 7 Eijkman-Oxford Clinical Research Unit, Jakarta, Indonesia; 8 Institute of Tropical Medicine Antwerp, Antwerp, Belgium; 9 Medicines for Malaria Venture (MMV), Geneva, Switzerland; 10 Instituto Leônidas & Maria Deane (Fiocruz), Manaus, Amazonas Brazil; 11 Fundação de Medicina Tropical Dr, Heitor Vieira Dourado, Manaus, Amazonas Brazil; 12 National Forum on Indonesia RBM/National Forum on Gebrak Malaria, Jakarta, Indonesia; 13 Universitas Sumatera Utara, Medan, Indonesia; 14 Yayasan Pengembangan Kesehatan dan Masyarakat, Papua (YPKMP), Papua, Indonesia; 15 University of Indonesia, Jakarta, Indonesia; 16 Mahidol Oxford Clinical Research Unit (MORU), Bangkok, Thailand; 17 Malaria Atlas Project (MAP), Big Data Institute, University of Oxford, Oxford, UK; 18 National Center for Parasitology, Entomology and Malaria Control, Phnom Penh, Cambodia; 19 School of Public Health, National Institute of Public Health, Phnom Penh, Cambodia; 20 Microbiology Department, Tribhuvan University, Kathmandu, Nepal; 21 Entomology Branch, Division of Parasitic Diseases and Malaria, Centers for Disease Control and Prevention, Atlanta, USA; 22 President’s Malaria Initiative, Malaria Branch, Division of Parasitic Diseases and Malaria, Centers for Disease Control and Prevention, Atlanta, USA; 23 Global Health Group, University of California San Francisco, San Francisco, USA; 24 Disease Control Division, Ministry of Health, Putrajaya, Malaysia; 25 UNICEF Indonesia, Jakarta, Indonesia; 26 Liverpool School of Tropical Medicine, Liverpool, UK; 27 Directorate General of Health Services, Ministry of Health & Family Welfare Government of the People’s Republic of Bangladesh, Dhaka, Bangladesh; 28 Save The Children, Kathmandu, Nepal; 29 ISGlobal, Hospital Clínic-Universitat de Barcelona, Barcelona, Spain; 30 Intellectual Ventures Global Good Fund, Bellevue, USA; 31 The London School of Hygiene & Tropical Medicine (LSHTM), London, UK; 32 Australian Defence Force Malaria and Infectious Disease Institute, Enoggera, Australia; 33 Center of Malariology, Parasitology and Entomology, Communicable Diseases Control, Vientiane, Lao PDR; 34 Bill & Melinda Gates Foundation, Seattle, USA; 35 Indian Council of Medical Research, New Delhi, India; 36 Department of Health, National Centre for Disease Control & Prevention, Manila, Philippines; 37 Malaria Branch, Division of Parasitic Diseases and Malaria, Centers for Disease Control and Prevention, Atlanta, USA; 38 PATH, Seattle, USA; 39 National Institute of Parasitic Diseases, China CDC, Shanghai, China; 40 WHO Office, Jakarta, Indonesia; 41 ACTMalaria, Manila, Philippines; 42 Ministry of Health, National Malaria Control Program, Jakarta, Indonesia; 43 Tengchong Center for Disease Control and Prevention, Tengchong, China; 44 Walter and Eliza Hall Institute of Medical Research, Parkville, Australia; 45 Epidemiology & Disease Control Division, Department of Health Services, Ministry of Health and Population, Kathmandu, Nepal; 46 Papuan Health and Community Development Foundation, Timika, Indonesia; 47 Ministry of Health, Kathmandu, Nepal; 48 Division of Vectors and Parasitic Diseases, Korea Centers for Disease Control and Prevention, Seoul, South Korea; 49 Ministry of Health, National Malaria Control Programme, Port Mosby, Papua New Guinea; 50 QIMR Berghofer Medical Research Institute, Brisbane, Australia; 51 School of Medicine, Addis Ababa University, Addis Ababa, Ethiopia; 52 Ministry of Health, Malaria and Other Vector-Borne Diseases Control Program (MOVBDCP), Port Vila, Vanuatu; 53 National Institute of Malariology, Parasitology and Entomology (NIMPE), Hanoi, Vietnam; 54 Eijkman Institute for Molecular Biology, Jakarta, Indonesia; 55 Jiangsu Institute of Parasitic Diseases, Wuxi, China; 56 WHO Collaborative Centre for Research and Training of Malaria Elimination, Wuxi, China; 57 Tuberculosis-HIV Research Center Faculty of Medicine, Universitas Padjadjaran, Bandung, Indonesia; 58 Radboud University Nijmegen, Nijmegen, The Netherlands; 59 Department of Medicine at the Doherty Institute, University of Melbourne, Melbourne, Australia; 60 Mahidol Vivax Research Unit, Faculty of Tropical Medicine, Bangok, Thailand; 61 National Vector Borne Disease Control Programme Directorate General of Health Services Ministry of Health & Family Welfare, New Delhi, India; 62 WorldWide Antimalarial Resistance Network (WWARN), Oxford, UK; 63 University of Washington, Seattle, WA USA; 64 Family Health Directorate, Ministry of Health, Jakarta, Indonesia; 65 Medicine Department, Medical College Kolkata, Kolkata, India; 66 Kenya Medical Research Institute (KEMRI) Centre for Global Health Research, Kisumu, Kenya; 67 Family Health Services, Ministry of Health, Port Mosby, Papua New Guinea; 68 Freeport Indonesia, Kuala Kencana, Indonesia; 69 Ethiopian Public Health Institute (EPHI), Addis Ababa, Ethiopia; 70 Independent Consultant, Bangkok, Thailand; 71 Yonsei University College of Medicine, Seoul, South Korea; 72 Program and Information Department, Directorate General of Disease Prevention and Control, Jakarta, Indonesia; 73 University of Gadjah Mada, Yogyakarta, Indonesia

**Keywords:** Vivax malaria, *Plasmodium vivax*, Adherence, Effectiveness, Efficacy, Radical cure, Primaquine, APMEN

## Abstract

The goal to eliminate malaria from the Asia-Pacific by 2030 will require the safe and widespread delivery of effective radical cure of malaria. In October 2017, the Asia Pacific Malaria Elimination Network Vivax Working Group met to discuss the impediments to primaquine (PQ) radical cure, how these can be overcome and the methodological difficulties in assessing clinical effectiveness of radical cure. The salient discussions of this meeting which involved 110 representatives from 18 partner countries and 21 institutional partner organizations are reported. Context specific strategies to improve adherence are needed to increase understanding and awareness of PQ within affected communities; these must include education and health promotion programs. Lessons learned from other disease programs highlight that a package of approaches has the greatest potential to change patient and prescriber habits, however optimizing the components of this approach and quantifying their effectiveness is challenging. In a trial setting, the reactivity of participants results in patients altering their behaviour and creates inherent bias. Although bias can be reduced by integrating data collection into the routine health care and surveillance systems, this comes at a cost of decreasing the detection of clinical outcomes. Measuring adherence and the factors that relate to it, also requires an in-depth understanding of the context and the underlying sociocultural logic that supports it. Reaching the elimination goal will require innovative approaches to improve radical cure for vivax malaria, as well as the methods to evaluate its effectiveness.

## Background

In November 2014 the governments of the Asia-Pacific nations reconfirmed their commitment to the regional malaria elimination by 2030 [1]. Over the last decade major gains in malaria control have been made, but these successes have been far less apparent for *Plasmodium vivax* than for *Plasmodium falciparum*. One of the greatest challenges in achieving the ambitious goal of malaria elimination is widespread implementation of the effective radical cure of malaria, in which all stages of the parasite are targeted. The propensity of *P. vivax* to form dormant liver stages (hypnozoites) and to recur, requires the provision of treatment of both the blood and liver stages of the parasite [2]. Currently, the only widely available drug to eliminate hypnozoites from the human host is primaquine (PQ). The World Health Organization (WHO) treatment guidelines recommend that PQ is administered over 14 days to reduce the risk of severe haemolysis [3]. Tafenoquine (TQ), another investigational 8-aminoquinoline compound currently under review by US Food and Drug Administration and the Australian Therapeutic Goods Administration, has a significantly longer half-life than PQ and, therefore, can be administered as a single dose regimen. However, the prolonged drug concentrations raise concerns over its potential to cause sustained haemolysis in G6PD intermediate and deficient individuals, highlighting the need for appropriate G6PD screening.

When supervised, a 14-day regimen of PQ can reduce the risk of recurrent *P. vivax* infection by more than 85% [4, 5]. However in most clinical scenarios daily supervision of a prolonged treatment regimen is not feasible. When drug administration is not supervised the clinical effectiveness of a 14-day course of PQ can be compromised severely [5–9]. In a recent large-scale observational study of 68,000 patients with vivax malaria in Papua, Indonesia, the effectiveness of unsupervised PQ was estimated to be at best 12% [10]. Whilst adherence to a 14 days regimen can be mitigated by reducing the duration of treatment [11], even an unsupervised 7 days regimen has been shown to be suboptimal [12]. Radical cure with a single dose of TQ will overcome many of the issues of adherence, however the challenges of ensuring concomitant G6PD testing will ensure that in many locations PQ regimens will still be the only available treatment option. Thus there is an urgent need to understand the determinants of adherence and to develop innovative approaches to improve anti-malarial effectiveness in malaria endemic areas. Investigation, rationalization and validation of novel interventions to improve adherence, requires measuring clinical effectiveness and this also poses specific challenges above and beyond conventional efficacy clinical trials.

As part of the ongoing collaborative efforts towards the elimination of malaria in the Asia-Pacific region, the annual meeting of the Vivax Working Group (VxWG) of the Asia-Pacific Malaria Elimination Network (APMEN) [13] was convened in Bali, Indonesia, in October 2017. The meeting was attended by 110 representatives from 18 partner countries and 21 institutional partner organizations. Data were presented on the challenges of adherence to antimalarial treatment regimens, lessons from the management of other infectious diseases, and clinical trials methodology. In addition two round table discussions were held to discuss (i) whether adherence to PQ radical cure was an issue, and if so how this could be overcome and (ii) methodological challenges in assessing anti-malarial efficacy and effectiveness of *P. vivax* infection. The specific questions posed to the groups are listed in Tables 1 and 2. The key discussion points are summarized into 4 categories: (i) impediments to PQ adherence at the level of the patient and health system, and suitable strategies to overcome these, (ii) tools and strategies that can be used from other disease programmes to improve adherence, (iii) measuring the effectiveness of PQ radical cure, and (iv) increasing the understanding of effectiveness and adherence using mixed methods research.

**Table 1:** Questions posed to participants for the first round table discussion

What are the main impediments of adherence from a patient perspective?
What are the main impediments of adherence from health care system perspective?
How can adherence be improved on the patient level?
How can adherence be improved on a health care system level?
Can methods from other disease programs be applied to malaria?
How can adherence to radical cure guidelines be improved?
How and what methods to increase adherence can be integration into current infrastructure?
What research is needed to better inform programs to improve adherence?

**Table 2:** Questions posed to participants for the second round table discussion

Quantifying effectiveness: what outcome measures are needed?
Quantifying effectiveness: how can observer biasbe minimized?
How can adherence be assessed in study setting/real life?
What should be priorities in qualitative surveys around adherence?
What public health interventions are needed to increase effectiveness of PQ?

## Topic 1: Impediments to PQ adherence at the level of the patient and health system, and suitable strategies to overcome these

Vivax malaria is often perceived as a benign disease and there is a lack of understanding and awareness regarding the benefits of radical cure; this constitutes the main impediment for PQ adherence at the patient level. There is a disconnect with the treatment of an acute febrile illness and the subsequent high risk of recurrent episodes of malaria, which are often regarded as new infections. Adherence to medication is influenced by a combination of social representation of medicines, such as the cultural and social meanings attributed to drugs and the cultural constructions of certain brand names [14, 15].

The beliefs among healthcare workers and policy makers were also regarded as being crucial, and these result in low rates of prescription, even when PQ is part of local and national treatment guidelines [16]. The perceived lack of benefit of radical cure is compounded in some locations by concerns regarding the potential side-effects of PQ, particularly the risk of haemolysis. Concerns regarding haemolysis and PQ toxicity, were apparent particularly in areas where G6PD deficiency could not be diagnosed reliably [16]. The lack of clear protocols to monitor and report haemolytic events after PQ treatment were mentioned as additional concerns. The acute febrile illness of vivax malaria is treated with standard schizontocidal drugs, but the additional prescription of PQ for the prevention of future recurrent infections is often regarded as being of secondary importance. Hence, concerns regarding potential adverse effects and the lack of immediate benefits, influence prescribing habits.

The need for better staff training and increased awareness about the individual and community benefits of radical cure were identified as a key strategy to overcome poor adherence by both the patient and the health provider. Participants highlighted the need for context specific strategies to improve adherence, including education and health promotion programmes aimed at increasing understanding and awareness within affected communities. Other interventions perceived as potentially beneficial included more intensive patient monitoring, which could be integrated with other disease programmes or home visits. This would reassure clinicians that if adverse events occurred they would be identified and managed promptly. Incentives could be devised to encourage providers to prescribe PQ and intermittent reminders could be provided to patients through mobile phone technologies to prompt them to continue treatment after their symptoms had abated.

Additional points raised during the discussion, included involving the private health sector in adherence monitoring, improved access to quality drugs, robust supply chains and the need for paediatric PQ formulations. Packaging aids such as blister packs or prepackaging compared to bulk package as well as pictorial inserts as suggested previously [17] were also deemed to be potential strategies (Table 3).

**Table 3:** Solutions to improve adherence to PQ

Main problem	Suitable interventions
Low patients adherence to PQ	Increase awareness among the general patient population about the benefits or radical cure through education and health promotion programs (health talks) and individual patients counseling Increased patient monitoring integrated into other disease programs, through home visits, incentives, pill boxes or automated reminders via text messages Packaging aids including pictorial inserts Better PQ formulations for children
Low provider adherence to PQ	Better staff training to increase awareness among staff about the benefits or radical cure and how to discuss these with patients Involvement of private sector in adherence monitoring. Improved access to quality drugs

## Topic 2: Tools and strategies used in other disease programs that can improve PQ adherence

Experiences from other disease programmes highlight the importance of adopting multiple approaches to improving adherence to treatment. A combination of efforts is needed and this should involve a multidisciplinary team of healthcare professionals (social workers, doctors, nurses, and counselors), patients (including close family members and carers) and the community (religious centres, community health organizations/agents and activists) [18]. Patient education and counseling were considered to be critical for all approaches, since both have been shown to improve patient-healthcare provider relationship, and provision of individualized and patient-centered care [19].

Education and counseling can be achieved at an individual level or at a family/group level. These approaches should include education about clinical consequences of vivax malaria, its propensity to recur and its treatment. Simple and pictorial stories and pamphlets can be used to increase patients’ competencies and knowledge. Health talks to the general public attending the hospital or health centre to disseminate information about malaria and suitable preventive treatments were considered feasible. The provision of easy and low cost adherence aids can help patients remember their medications and the order and timing at which they should be taken. For instance, a pill box, in which doses are divided up by days or a pill taking table, can be filled in by the patient or someone supporting their treatment; these remind patients to take their medication, which tablets have already been taken and which tablets are still remaining. Alternative options include setting alarm clocks or mobile phone alarms to remind patients when to take the medication or matching pill taking to the patient’s daily activities (e.g. before, or after a meal, or prior to a daily activity) [20]. Treatment support outside the health centre can be provided by either family members or community health agents. In Nepal a patient-responsive approach including both of these strategies has been shown to improve the adherence to tuberculosis treatment [21].

An integrated package of interventions was generally perceived to be more useful than single intervention. Furthermore the most suitable interventions are likely to be context specific. Thorough evaluation of the efficacy and effectiveness of new approaches including cost–benefit analyses is needed for National Malaria Control Programmes (NMCPs) to make informed decisions on optimal strategies.

## Topic 3: Quantifying PQ effectiveness to optimize suitable interventions

Most anti-malarial clinical trials measure treatment efficacy, which refers to the ability of a regimen to achieve cure in an ideal scenario. Effectiveness is the extent to which a drug regimen achieves its intended effect in the real world setting. To determine effectiveness one needs to document the clinical outcome, such as recurrent episodes of malaria or associated anaemia, but in such a way as to minimize the influence on patient and prescriber behavior. Two questions were posed to participants: how can adherence be measured reliably and how reactivity can be minimized without compromising the trial’s integrity?

Typical measures of adherence include self-reporting, pill count and biological assays. Self-reporting is used widely since it is relatively easy and cheap to implement, however it is also subjective, highly dependent on how questions are asked, prone to recall and social desirability bias [22]. Using text messaging as a self-reporting tool was discussed as a potential way to reduce social desirability bias. Computer administration has been shown to increase reporting levels of sensitive questions compared to interviewer-based administration of questionnaires [22–24]. However interactive text-messaging relies on significant infrastructure and few studies have estimated its reliability [22].

Pill counts are also simple to implement using either manual counting or an electronic pill cap [25, 26]. Manual pill counts are usually undertaken during follow up visits at the health facility, with patients asked to bring the empty pill box or empty blister packs. However, this method is also prone to potential bias, since patients are aware that their compliance is being assessed. Home visits or unannounced calls to patients were discussed as a more appropriate to estimate true adherence, although this raises ethical considerations requiring prior consent when enrolled into the study [27, 28]. Electronic pill caps, were considered the reference standard of indirect adherence measures. These record the opening of the pill box, the underlying assumption being that bottle opening represents medication intake, however this level of complexity comes at a greater financial cost [29].

Biological assays offer another measure of adherence. Plasma drug concentrations can be quantified to demonstrate that drug doses were ingested, however these are confounded by the actual dose prescribed, its timing with respect to sampling, gastrointestinal absorption, metabolism and elimination. Furthermore such approaches require invasive sampling from either capillary or venous blood samples, and involve significant infrastructure to ensure adequate sample storage and analysis. PQ is metabolized rapidly with a half-life of less than 6 h, so may be undetected within 24 h of administration [30]. Its metabolite, carboxy-PQ, is more slowly eliminated and accumulates over the course of a 14 day course and may therefore be a more useful measure of adherence [30]. Correlation of carboxy-PQ plasma concentrations and adherence requires knowledge of the normal pharmacokinetic profile of the drug when administered under supervision in a variety of populations; however this has yet to be defined. Sparse sampling similar to population pharmacokinetic studies with a probability score as outcome measure was discussed as a potential solution, but such approaches need to be validated in carefully controlled settings.

Quantifying methaemoglobin (Met-Hb) concentrations is another potential proxy measure of adherence. Met-Hb concentrations are elevated following PQ administration since the drug induces oxidative stress on red blood cells resulting in oxidization of the ferrous iron in the haem group [31]. Met-Hb can be measured non-invasively using a finger probe to measure arterial oxygen-Hb saturation. Similar to the interpretation of blood drug concentrations, the normal variation of Met-Hb following supervised PQ therapy needs to be defined. Furthermore Met-Hb concentration may be elevated due to other reasons besides PQ administration and thus validation of this approach is needed. A study in Bangladesh showed no clear correlation between Met-Hb levels and pill counts [32] and recent data from Brazil also indicated no correlation between the plasma PQ concentrations or total dose and Met-Hb levels [33], although further clinical studies are underway to explore this further.

Anti-malarial clinical efficacy trials require patient observation to capture the relevant study outcomes. However the act of observing people alters their behaviour, a phenomenon often described as reactivity or observation bias in psychological research and behavioural science [34–36]. Patients enrolled into a study often do not behave as they would in real life, just because they are aware that they are in a study, are being observed and receiving a therapeutic intervention. Comparative efficacy trials can mitigate this potential bias by “blinding” treatment so that either the patient and/or the clinician are unaware of the treatment arm they are receiving. Although this reduces behavioural bias between treatment arms being compared, it does not overcome changes in behaviour inherent in being under scrutiny. Hence adherence in intervention and control groups often do not reflect reality. Reactivity can be reduced by minimising observation or by using less obtrusive measures, such as unannounced pill counts as discussed previously [28]. Early enrolment and consent of a cohort before malaria infection and treatment occurs, using a cluster design was suggested as a way to reduce reactivity as the effect can be expected to wane over time [36]. Studies that are integrated into the routine health care systems rather than stand-alone trials provide an alternative way of capturing clinical outcomes under normal clinical practice, although these are confounded by a lack of a control arm, residual confounding and attrition bias [10].

## Topic 4: Increasing our understanding of effectiveness and adherence using mixed methods research

Reducing reactivity alone is insufficient to ensure robust measures of effectiveness or the effect of novel interventions targeting adherence. Measuring adherence and the factors that relate to it requires an in-depth understanding of the context and the underlying sociocultural logic that underlie it [37]. This requires a better understanding of the implicit heterogeneity in behaviour, cultural diversity and variability in social structural conditions underlying adherence.

Qualitative methods provide an important approach that can inform the optimal design of quantitative adherence studies. Contextual information can facilitate the selection processes to minimize bias, determine specific sub-groups at risk of being excluded, inform the operationalization of concepts to be measured, and aid the interpretation of the results. Suitable qualitative methods such as ethnography constitute effective tools to provide an in-depth understanding of the factors contributing to adherence. These data should be central to the design of appropriate intervention strategies to impact on behaviour and clinical outcomes. Quantitative measures of adherence need to consider the sociocultural specificities and thus are likely to vary considerably between different endemic settings. It is unlikely that a single approach can be proposed for suitable interventions or the design of an effectiveness trial that is widely applicable across a wide range of environments. However further exploration may reveal certain common elements that can be applied universally.

Various additional constraints for the delivery of effective radical cure were mentioned and considered to require a more in-depth and locally relevant understanding. These included human mobility and how this affects adherence and follow up, and patients’ flexible health seeking itineraries and therapeutic choices, such as the private, traditional sectors and home treatment.

## Conclusions

In the light of the 2030 elimination goal, the delivery of effective radical cure for malaria is a high priority for the vivax endemic areas of the Asia-Pacific Region, Horn of Africa and South and Meso-Americas. The design and evaluation of appropriate interventions to improve effectiveness of PQ treatment, as well as lessons learned from other disease programmes must be taken into account and the methodological challenges addressed.
